# Obstacles and appeal of environmental taxation: Insights from sub-Saharan Africa

**DOI:** 10.1016/j.envdev.2024.101037

**Published:** 2024-09

**Authors:** Giovanni Occhiali

**Affiliations:** Institute of Development Studies (IDS) and International Centre for Tax and Development (ICTD), United Kingdom

**Keywords:** Environmental taxes, Climate taxes, Sub-Saharan Africa, Tax administration

## Abstract

Increasing the slow pace of adoption of environmental taxes across low-income countries has become a significant priority among international financial institutions, multilateral development banks, and international donors. Yet little is known about the practical institutional, administrative, and political obstacles that have led to their slow implementation and how they can be made more appealing, especially across sub-Saharan Africa. Based on a review of the literature and 16 in-depth interviews with ministries of finance, revenue authorities, and other government representatives across six African countries, this paper provides exploratory evidence of these stakeholders' view about environmental taxes deployment in their countries' context. By increasing the visibility of these policymakers’ opinions and priorities, this study aims to individuate areas in which further research can provide support for the introduction of environmental fiscal measures. While there are differences across the countries covered, a lack of data and analytical capacity to develop effective environmental taxes is a common theme, as well as the historical prioritisation of their revenue mobilisation capacity over their environmental impact. A great variety of government actors with a mandate over natural resources, often with competing policy priorities, coupled with a lack of coordination fora, has also impeded the harmonisation of the environmental charges they levy. These measures are also often perceived to be regressive and to pose an obstacle to industrial development, lowering their appeal, given that poverty reduction and employment creation are an overarching priority. Nonetheless, support for introducing specific environmental tax measures exists across the population and policymakers, especially if their revenue can be earmarked for environmental purposes.

## Highlights

• Little evidence is currently available on the obstacles to a wider deployment of environmental taxes in Sub-Saharan Africa.
• The data required to devise effective environmental tax policies and the capacity of modelling it are usually lacking.
• A variety of government actors have competing mandates on environmental resources and coordination is complex.
• Environmental taxes are perceived as regressive and a hindrance to industrial development and poverty reduction.
• Support can be mustered for taxes addressing localised environmental issues, especially if their revenue is earmarked.

## 1 Introduction

In the last few years, there has been an increasing push for the introduction of environmental taxes (ETs) 1 across the world, that is, charges on air pollutants emitted through industrial production or vehicle use, levies on household waste, or taxes on energy use. These fiscal measures are seen as instrumental to both increasing revenue mobilisation and tackling environmental issues, and have proved useful to reduce greenhouse gases emission and improve energy efficiency in both the European Union specifically (Lutz and Meyer 2010; Onofrei et al., 2017; Li et al., 2023) and industrialised countries more generally (Murray and Rivers 2015; Mardones and Mena 2020; Farooq et al., 2023). In themselves, discussions about the usefulness of ETs are not new – they have been studied since at least the 1990s and were implemented throughout Europe and North America soon afterwards (Shah and Larsen 1992; Smith 1993; McMorran and Nellor 1994). The novelty lies in the type of countries where it is now being suggested that they seriously consider introducing ETs, many of which are low- or lower-middle-income countries (henceforth LICs for simplicity). Indeed, LICs are facing increasing financial pressures while concurrently suffering from severe environmental degradation, so implementing measures that could tackle both at once seems an obvious choice. Consequently, it is unsurprising to learn that there is a general consensus amongst international financial institutions, multilateral development banks, and international donors that introducing ETs should be widespread (IMF 2019; Pigato 2019). In its most ambitious form, implementing these measures should be part of wider environmental fiscal reform strategies, which would shift tax pressure away from capital and labour, hence also contributing to increasing growth rates by liberating productive forces (OECD 2005; Boyd et al., 2005; Pigato 2019; IMF 2019).

Yet, there is a dearth of empirical evidence concerning which ETs are already part of LICs’ policy package, which are seen as a priority, whether they are actually implemented and with what impact, and if not, what the main challenges to their implementation are (Carattini 2022; Cottrell et al., 2023; Keen 2024). This is due to several different reasons. First, there are a variety of instruments that could potentially qualify, from those targeting fossil fuel extraction or consumption to those levied on the exploitation of natural resources and the importation of used cars (Cottrell and Falcão 2018). Second, there is still some debate about whether only measures with a primarily environmental objective should qualify as ETs or if those targeting an environmentally related base but exclusively aiming to mobilise revenue should also count (Cottrell and Falcão 2018; ATAF 2021). Third, it is extremely difficult to gather information on which ETs measures are practically applied across LICs and what they contribute to the state purse (Cottrell et al., 2023). Hence, the vast majority of existing studies on the deployment of ETs have focused on high-income countries (HICs) or upper-middle-income countries for which more data is available. While some of the original flagship reports from various institutions on the topic also covered some lower-middle-income countries (OECD 2005; Boyd et al., 2005), the information in these studies is likely outdated now.

This lack of evidence is at its starkest for sub-Saharan Africa (SSA). The few existing studies on the region (McMorran and Nellor 1994; Abaza and Rietbergen-McGracken 1998; Resnick et al., 2012; Slunge and Sterner 2012; Belletti 2020) indicate that most measures which could qualify as ETs seem to have been introduced more with revenue than with environmental goals in mind. These studies also mention that administrative complexities, issues with institutional coordination, and concerns about the political consequences of their deployment are all significant obstacles to the wider introduction of ETs but do not provide much explanation of their actual nature. While all these obstacles have historically been significant in HICs (OECD 2005; Boyd et al., 2005), there are good reasons to believe they will manifest differently in LICs, given the wide divergences in economic, institutional, and social characteristics. Furthermore, virtually nothing is actually known about these measures’ political appeal among both policymakers and the wider public.

This paper aims to provide exploratory evidence to fill this gap by discussing the main administrative and political obstacles to the deployment of ETs, as well as their political appeal, across various low- and lower-middle-income countries in SSA. The evidence presented originates from 13 in-depth interviews carried out in June and July of 2022, both online and in-person, with managerial staff from revenue authorities and ministries of finance in eSwatini, Ethiopia, Rwanda, Uganda, and Sierra Leone, as well as with two members of the Nigerian Governors Forum working on climate issues and an international Public Financial Management (PFM) consultant in Uganda.^2^ These interviews investigated the most pressing environmental issues in each country, whether solving them was a government priority and if any environmental fiscal measure was in place to tackle them. The characteristics of administrative, institutional, or political obstacles to the introduction of other ETS were also discussed, and so was their domestic political appeal. This study intends to highlight the views of an important group of government stakeholders whose opinion has so far been under-represented in the literature and in international policy-debates, with the intention of identifying areas in which further research can support the deployment of ETs.

A series of limitations regarding the representativeness of the views discussed in this piece must be acknowledged. First, the interviewees for the study ail from only 6 of the 46^3^ to 48^4^ countries normally considered to be part of SSA, which were selected due to their longstanding research collaborations with the author's centre. While their stakeholders' perceptions and experiences are not necessarily representative of those of their peers in the whole region, there is enough diversity in the countries covered, be that in geographical, institutional or socio-economic terms, for the study to speak to a variety of different contexts. Second, while SSA is probably best described as region where pluralist legal traditions, mixing aspects of common, civil and traditional or customary law, is nowadays predominant (Kischel 2019), there have long been debates about the importance of different colonially inherited legal systems on each country socio-economic development (Joireman 2001; Selassie 2003; Samuels 2019, Michaels, 2021). To the extent that colonial legacies still influence modern policy processes, then the sample is skewed towards former British colonies, in which common law constitutes the skeleton of contemporary legal systems. Future research might focus on countries in Francophone and Lusophone Africa where civil law represents instead the legal backbone. Finally, as all study interviewees are civil servants or consultants in the public administration, the views of civil society and private sector actors on the state of ETs deployment in their countries is not represented in the study. This is by no means due to a lack of relevance of these stakeholders in the tax reform process, as recent research highlights the role that especially civil society organisations play in improving the legibility and transparency of the tax system (Sharp et al., 2019; van den Boogaard et al., 2021). However, as these organisations usually focus on analysing existing tax policies and on raising awareness of their connected obligations, understanding the opinions of those in charge of creating these policies in the first place seemed like the correct starting point. Future studies carried out after more ETs are actually in place should though also include the perspectives of these important actors.

Regarding the study results, although there are obviously differences among the countries examined, the interviews also revealed various similarities. Firstly, all the abovementioned countries are struggling with deforestation, poor waste management, and traffic pollution in urban areas, and they see these as issues that fiscal policies could contribute to addressing. While the connected environmental degradation is confirmed to be an increasingly salient political issue, it still represents a significantly lower priority than poverty reduction and employment creation. Indeed, there are fears that solving the former could be at the latter's expense, as focusing on environmental protection could hinder industrial development by slowing the influx of Foreign Direct Investment (FDI). The existing political support might be sufficient to introduce discrete tax measures tackling localised issues in different countries, but the ongoing cost-of-living crisis makes wider reform less appealing, and earmarking of ETs revenue could help garner public backing.

Secondly, the few ETs in place have mostly been introduced for revenue reasons and are perceived to be underperforming, although seldom subjected to rigorous evaluation. Environmental fees and charges introduced by environmental protection agencies or various line ministries are more frequent than ETs proper, and ministries of finance and revenue authorities are scarcely involved in their development or administration.

Thirdly, the main obstacles to a wider deployment of ETs are the lack of both environmental data and the capacity to analyse what is present, as well as a lack of coordination fora for the various ministries and agencies with a mandate over environmental resources, so that policy approaches remain fragmented when not altogether competitive. However, this is an area that has seen recent developments with the creation of ministries of environment, but they have not been active long enough to judge whether they are having a positive impact.

Fourthly, despite having received by far the most attention from the international community, carbon taxes were seldom mentioned independently by the officials interviewed and were never identified as a priority area.^5^ On the contrary, in countries where they had explicitly been considered, their implementation was always shelved due to both considerations of technical capacity and a lack of perceived immediate benefits. However, it must be noted that the interviews predate the adoption of the carbon border adjustment mechanism by the European Union, so this picture might have changed since.

The paper proceeds as follows. The next section presents a brief overview of the literature on environmental taxation, describing areas that have received the most attention in academic research and what is known about SSA countries. Section 2 then moves on to discussing the main environmental issues in the countries covered according to our interviewees, which fiscal measures exist to support tackling them and whether they are perceived as being effective. Section 3 describes the more prominent administrative, institutional, and political obstacles, while Section 4 discusses these measures' main domestic appeals. Finally, Section 5 provides a conclusion and potential next steps.

## 2 Literature review

ETs are a form of Pigouvian tax: they are charges on the environmental externalities derived from the production or consumption of goods and services and applied to correct market prices, and they force producers or consumers to internalise these costs (Pigou 1920). However, it is very complex to determine the exact societal cost of many environmental externalities; it is, therefore, more common to first define their desired level – e.g. the maximum amount of wastewater that should be produced from leather tanning – and then determine a tax that will lead to its achievement (Spratt 2012). Often, this is done by targeting specific production inputs as tax bases rather than the externalities themselves – e.g. the chemicals involved in leather tanning rather than the pollution they produce – with the loss in economic efficiency arising from this strategy counterbalanced by easier administrative implementation (Panayotou 1994; Spratt 2012).

Major international institutions have promoted greening the tax system by introducing environmental fiscal reforms since the 1990s (Shah and Larsen 1992; Smith 1993; McMorran and Nellor 1994), and continue do so in the current days (Pigato 2019; Carattini 2022; World Bank 2022). While the capacity of ETs to mobilise substantial revenue was debated from the beginning, with some doubting it (Muzondo et al. 1990) and some more convinced of their potential (O'Connor and Turnham 1992), there is a consensus that they can help tackle environmental issues while generating at least some revenue (OECD 2005; Boyd et al., 2005; Pigato 2019; Keen 2024). The calls to introduce ETs were heeded by HICs from the very beginning, but deployment across LICs was much slower (O'Connor and Turnham 1992).

This was partially because ETs often tend to be regressive in contexts where low-income households rely more on natural resources, consume a disproportionate amount of polluting goods, and are less likely to enjoy environmental improvements (Panayoutou 1994) – a characteristic of many LICs. The still limited experience with ET instruments in HICs, reducing the availability of lessons learnt, coupled with an extensive prevalence of communal and customary property rights tenures on natural resources and a lack of technical expertise in ecological management in LICs were also seen as contributing to the slow adoption pace (Panayoutou 1994).

Precisely which measures were more appropriate for LICs contexts was – and still is – an object of debate. Fuel and transport taxes are usually seen as having good potential, as they are relatively easy to administer and have good revenue potential (Heady 2002), although this can often be eaten up by the provision of fuel subsidies. Indeed, these taxes have been very commonly applied across a range of countries (Cottrel and Falcao 2018; Cottrell et al., 2023; Keen 2024), including Mauritius (Parry 2011; Slunge and Sterner 2012), South Africa and Ethiopia (Slunge and Sterner 2012), Barbados (Moore et al., 2014), Kenya (UNEP 2015a); and Mozambique (UNEP 2015b). However, there is potential to increase their positive impact on the environment, as most of these measures have been introduced to raise revenue rather than to tackle transport-related externalities (Granger et al., 2021), and the example of South Africa shows that they can indeed have an impact (Nkosi et al., 2021). Forestry taxes have also received quite a lot of attention in the literature, as emissions from land use, land-use change and forestry represent the main source of greenhouse gas emissions on the African continent (AfDB 2020), and fiscal instruments can support the fight against deforestation (World Bank 2021, Keen 2024). Despite what was felt to be significant revenue and environmental potential, various analyses suggest that their actual impact has been very limited (Leuth et al. 2000; OECD 2005), owing to various compliance and administrative issues, as well as the extensive corruption plaguing the sector, which often remain unaddressed (Ross 2001; Hansen and Lund 2011; Cerutti et al., 2013; Carlsen and Hansen 2014; Hoare and Uehara, 2022; Occhiali and Falade 2023). Taxes on single-use plastic have also been adopted by different countries on the continent, although with apparently limited environmental impact (Keen 2024, or see Dikgang et al., 2012 for South Africa), with bans and other non-market-based approaches to their management remaining widespread (Adam et al., 2020). Given the existence of a variety of tax instruments which can help addressing waste-related externalities (Matheson 2022), there might be further measures which would be worth taking into consideration.

Indeed, it is highly likely that political economy considerations are one of the main reasons for the slow introduction of ETs across many LICs. As such, they have been receiving attention for quite some time (Boyd et al., 2005; OECD 2005). Political economy considerations are relevant because different stakeholders rely on natural resources for different reasons and will, therefore, conceptualise them accordingly. Using forests as an example, they represent a source of energy and potential agricultural land for adjacent communities (Tegegne et al., 2016), a source of rent for politicians involved in patron-client relationships in the allocation of timber rights (Hansen and Lund 2011; Carlsen and Hansen 2014), and carbon storage for the international community (Leach and Scoones 2013). Hence, any policy that directly impacts the cost or ease of forest exploitation will elicit different and potentially contrary responses from users and policymakers depending on which view they align with. This situation is not unique to forestry: similar considerations can be made for commercial fisheries (Occhiali 2023), for the exploitation of various minerals, and for all manufacturing activities giving rise to pollution (Boyd et al., 2005; OECD 2005, Pigato 2019, Cerutti et al., 2013).

Extensive sensitisation about the reasons to introduce ETs, as well as some revenue recycling, could help decrease public opposition, as has been shown by the various and often unsuccessful efforts to remove or reduce fossil fuel subsidies across many LICs (Vagliasindi 2013; Fay et al., 2015; UNECA 2016). As mentioned above, the distributional impact of ETs will have to be taken into account across LICs, and especially across SSA, where authors pointed out that green growth strategies risk increasing poverty if they are not balanced by redistributive policies (Resnick et al., 2012). While ETs are not inherently regressive,^6^ even a progressive tax can negatively impact lower-income groups in the absence of effective redistributive policies, which are often lacking on the continent (Odusola 2017). However, many LICs will have to develop their administrative capacity to effectively deploy and enforce carbon taxation (Krogstrup and Oman 2019), a consideration that likely stands true for a variety of other ETs (Keen 2024).

While, as it was shown, the scope for introducing ETs is quite broad, it is fair to say that since the late 2000s, the focus of most academic and international institutions has been on carbon taxes, which are seen as the most promising instrument to decrease carbon emissions (IMF 2008; OECD 2009; de Mooij et al., 2012; Parry et al., 2022). Indeed, especially in HICs, carbon taxes can help to address emissions at their source, while potentially raising substantial revenue at a minimal welfare cost (OECD 2005; Murray and Rivers 2015; Parry et al., 2022). The funds generated from carbon taxes could then be used to reduce fiscal pressure on capital and labour (Panayoutou 1994; Norregaard and Reppelin-Hill 2000; OECD 2008; Pigato 2019). Alternatively, there have also been those proposing to recycle some carbon tax revenue from HICs to address environmental issues in LICs, as introducing said taxes in the latter would do little more than avoid carbon leakages (de Mooij et al., 2012). However, it must be noted that since the late 2010s, there have been increasing calls for carbon taxes to be widely applied across LICs from institutions as diverse as the UN,^7^ the OECD,^8^ the IMF,^9^ the Institute for Fiscal Studies,^10^ and Brookings.^11^ While more critical voices also exist (KAS, 2020; Mager and Chaparro 2023), the recent introduction of a carbon border adjustment mechanism in the European Union might have significantly altered LICs incentives to seriously consider this measure. This adjustment mechanism would lead to charges based on the carbon content of imports to the European Union, competing with energy-intensive industries taxed under European law (Keen et al., 2021). While there currently are no in-depth studies of the potential impact of this mechanism on LICs, as the measure will only come into force in 2023, early analysis (UNCTAD 2021; Pleeck et al., 2022) shows that it might lead to significant costs across particular countries. Indeed, this measure might well represent LICs’ strongest incentive to consider carbon taxation – if someone is going to tax their carbon emissions, it might as well be them.

However, the example of South Africa, the only country in the continent that has currently introduced a carbon tax, shows how complex it can be to introduce these measures effectively: it took nine years to implement the carbon tax, which only became politically feasible after exempting 95 per cent of emissions (Baker 2022). Consequently, the tax has raised a paltry 0.11 per cent of total revenue in 2021–22, and although its impact on emissions has not been quantified, it is unlikely to be particularly significant. In fact, widespread discussion about carbon taxation across the continent might well be mostly due to the influence that international debates still have on fiscal policies in the continent, with revenue collection often used as benchmark for aid disbursement by donors (Moore 2020). Indeed, although diminished from past days (Adam and Bevan 2001), both the World Bank and the IMF still possess an influence on tax policy in LICs (Moore 2023), so it is certainly possible that this could extend to ETs strategies too, especially considering that LICs are currently dependent on international aid to satisfy their adaptation and mitigation need (UNEP 2023).

## 3 Environmental issues and existing measures to tackle them

Unsurprisingly, all the stakeholders interviewed recognised the existence of a variety of environmental issues, some of which were shared among multiple countries. To start with, deforestation was mentioned as a pressing issue in multiple instances,^12^ leading to more frequent and impactful flooding in rural areas, as well as more mudslides.^13^ Indeed, the three countries whose stakeholders brought this up have lost 8.7 per cent (Rwanda), 13 per cent (Uganda) and 35 per cent (Sierra Leone) of their forest cover since 2000 (Global Forest Watch 2023), with the interviewees mentioning that much of this is loss is due to charcoal production and the expansion of agricultural land.^14^

Another issue that arose frequently was that of pollution in urban areas, both from the increasing numbers of privately-owned vehicles, many of which are second-hand,^15^ and from poor waste management practices.^16^ While data about both of these topics is extremely scarce for SSA countries, what is available lends support to the perception of the stakeholders interviewed. For example, the WHO recommends an average PM2.5^17^ mean annual exposure of 5 μg/m3 (WHO 2021), well below the average levels between 2010 and 2019 of 34.3 μg/m3 in Ethiopia, 49.9 μg/m3 in Sierra Leone and 40.2 μg/m3 in Uganda (World Bank 2023). Similarly, the most recent Waste Management Outlook estimates that less than 42 per cent of waste generated in Ethiopia and Uganda is properly collected, with the equivalent figure being lower than 55 per cent in eSwatini and Sierra Leone (UNEP 2018). In particular countries, in addition to the urban pollution from both sources, one must add that from the growing manufacturing sector, which is foreseen to account for a growing share of emissions in the coming years.^18^ Despite the clear human health impact of these types of environmental pollution and degradation, it should also be noted that very few explicit considerations were made during the interviews on the link between environment and health.

While finding a solution to these issues is becoming more pressing in different countries, this is far from the main governmental priority. However, there is a recognition that the pace of action must accelerate.^19^ Indeed, some fiscal measures have been introduced in recent years as part of the policy package to address them. Various countries have introduced taxes or fees on plastic products to reduce waste generation,^20^ duties on used cars varying depending on the age of the vehicle imported are becoming more common,^21^ and there are examples of governments taking action to support reforestation, often with donor support.^22^ Nonetheless, the introduction of market-based instruments such as ETs or subsidies remains sparse, as the prevailing approach across most SSA remains that of command-and-control.^23^

However, measures that could qualify as ETs have been in place for much longer, although generally not contributing much revenue – 0.22 per cent of GDP in 2021 across the 17 countries reporting this information (ATAF 2023), and this aggregates over very different instruments. Stakeholders from all countries interviewed mentioned the existence of fuel levies, as well as a variety of charges levied on the extraction of both renewable and non-renewable resources, with the latter mostly managed by line ministries rather than by revenue authorities. Various stakeholders also recognised that environmental management or protection agencies (henceforth EMAs for simplicity) could also be levying a variety of fees but admitted that they did not have much information about them, as revenue authorities and ministries of finance are seldom involved in their setting.^24^ Finally, local government authorities such as city councils also have some revenue-raising capacity with regard to ETs, for example, through the introduction of waste taxes.^25^

However, what sets this second group of measures apart is that they are invariably administered to mostly raise revenue, even in cases where their introduction purposely had a mixed revenue/environmental objective.^26^ Interestingly, the opposite seems to apply when environmental subsidies – which are becoming more common to promote renewable energy adoption, silviculture, and waste management – are considered. In these cases, promoting environmentally sustainable practices is a much more apparent goal than simply promoting economic activities or employment.^27^ This is likely the case as there is currently a widespread lack of greener alternatives – such as public or electric transport, affordable gas stoves or proper waste disposal facilities – available in domestic markets, which makes relying on ETs as a behavioural tool more complex.^28^

Furthermore, as many existing measures are not directly managed by revenue authorities or ministries of finance, there is little knowledge of their impact, which is seldom rigorously assessed, and their actual revenue contribution.^29^ However, there is a perception that they have not been particularly useful in tackling existing environmental issues.^30^ This could be due to a variety of reasons. First, some of these measures are dated and contain no provisions for automatic updates of rates charged, which usually happen irregularly and in an ad-hoc way.^31^ Second, there is generally no provision for earmarking their revenue to tackle environmental issues, with all collection simply flowing to consolidated revenue funds.^32^ Third, some of these measures are weakly enforced, either because of frequent restructuring of the institutions overseeing their implementation, or simply for lack of political will.^33^

## 4 Institutional, administrative, and political obstacles

Despite their current ineffectiveness, most stakeholders interviewed thought that ETs could play a role in addressing existing issues, such as pollution from vehicles,^34^ deforestation,^35^ or emissions from the agricultural sector.^36^ However, as expected from the literature, a series of institutional, administrative, and political obstacles need to be addressed before ETs can be more effectively deployed.

The main institutional obstacle existing across all countries interviewed is simply the number of different ministries, departments, and agencies (MDAs) that have competing mandates – and revenue-raising capacity – on environmental assets and natural resources. These usually include the EMA, the ministry of agriculture, the ministry of environment – with either of the last two potentially overseeing natural resources, the ministry of tourism – which might have oversight over the EMA, the ministry of finance, and the revenue authority. Additionally, ministries of mines or national mineral agencies, energy and water regulators, institutions in charge of looking after specific natural bodies, such as forest reserves or lakes, and green investment funds could all also be involved in managing some aspects of natural resources or stake claim on the revenue generated from them. Moreover, this is before considering the case of federal states, such as Ethiopia or Nigeria, where many of these institutions might exist at the federal and state levels.

The reason why this policy fragmentation is an issue might not be self-evident. While it can be argued that line MDAs are better placed to first design and then collect fiscal charges on resources on which they have the remit, this is, in fact, not always the case. Firstly, remits are not always exclusive. For example, a non-gazetted forested area lying on an individually owned plot may incur charges from the forestry commission, the ministry of agriculture and the ministry of land, or activities in coastal areas could attract fees from both the ministry of tourism and the ministry of marine resources. Said charges might well be providing inconsistent – while not altogether opposite – incentives for the economic use of these areas, which has environmental consequences.

Secondly, many line MDAs are tasked with stewardship of particular resources rather than their direct economic valorisation. Granting them revenue-raising capacity might lead to adverse situations where conserving environmental resources becomes less of a priority than meeting revenue targets, as this could well be connected to their budget release (see Occhiali and Falade 2023 for an example on forestry management in Nigeria).

Thirdly, while line MDAs know the sector they oversee, they might not possess sufficient knowledge of fiscal instruments' behavioural and economic impact to set charges at an appropriate level. On the other hand, ministries of finance are better placed in all these respects – their policies should ideally provide an overarching and unified framework for the economic valorisation of different resources, and they have the skills to evaluate the economic impact of the measures they promote.

Despite this evident institutional complexity, fora for all existing MDAs to coordinate their actions and charges have historically been lacking, with inter-ministerial committees or ministries of the environment having been created only recently and in just a few countries.^37^ Hence, harmonising levies and policy objectives across MDAs has usually been a challenge, as different institutions might have conflicting ideas on how to develop specific areas (e.g. forest conservation or mono-crop plantation) and will try to raise revenue accordingly. Ad-hoc meetings during the budget process, one of the few instances in which almost all MDAs interact with each other on a yearly base, are not sufficient to resolve these differences.^38^ In countries that have recently created dedicated ministries or committees, what remains to be seen is whether these new bodies will be able to provide clear policy priorities and instructions to the many MDAs involved. The risk of simply having added another player to a context where every MDA brings its own different approach to the same issue and tries to protect its existing power is all too real.^39^ On the other hand, countries that are still lacking any coordination fora think that instituting one could help the government to have a proactive rather than reactive approach to environmental issues. However, some stakeholders doubt that the political will to establish one exists if environmental protection is seen as an obstacle to overcome.^40^

Another extremely frequent obstacle is the lack of both proper environmental data and the expertise required to analyse it across revenue authorities and ministries of finance.^41^ Some data and sufficient technical capacity are present across some EMAs,^42^ but there are also instances where, even in these institutions, the required technical skills to carry out their mandate are lacking.^43^ Even where the EMAs had the appropriate data and technical capacity, they still might not have the competencies required to be put in charge of developing ET frameworks. This is because ETs are inherently market instruments, and the command-and-control approach of EMAs often leads to a lack of appreciation of the economic consequences of their policies.^44^ Combining the capacities of EMAs, revenue authorities, and ministries of finance might be sufficient to develop particular ETs, but this leads back to issues of institutional coordination and objectives.^45^ Furthermore, even then, questions about the main priority of ETs – raising revenue or tackling environmental issues – and about which MDAs should be collecting the revenue would remain and would only be solved through political bargaining.^46^

There is a variety of data currently lacking across all countries to develop effective ETs. Firstly, industrial actors are generally not required to submit most of the information required to devise effective taxes on pollution, and there are significant doubts about whether they will have this information themselves.^47^ Given this situation, proxies from comparable countries have been used to develop policies such as plastic taxes, but this still gives room for practical differences across the industries to diminish policy effectiveness.^48^ Similarly, little is known about households' willingness to pay for different services, such as waste management, and without this information it is difficult to determine which tax rates are acceptable to the population, which is relevant to ensuring compliance.^49^ Data on renewable natural resource stocks, such as forests and fisheries, can also be lacking or outdated, again diminishing the likelihood of devising effective policies.^50^ Given this background, the most reasonable approach is for governments to proceed in areas for which some data is present across different MDAs, such as developing import tariffs on used cars based on data from road safety authorities.^51^

One point on which there was some significant difference across the stakeholders interviewed was whether administering ETs would be more complicated than other taxes.^52^ Some thought that the administrative systems already in place could easily be expanded to include a more significant share of ETs as long as they are structurally similar to other existing taxes.^53^ Others maintained that by their very nature, ETs will require investments in data collection and reporting at the firm level and in monitoring capacity at the government level, which will make them more costly to both comply with and administer.^54^ Furthermore, it could well be that firms will be required to report environmental information to one MDA, but then another one will be tasked with collection, which again could give rise to friction at the government level.^55^ Given how significant collecting the right information will be, appropriate time should be dedicated to striking the right balance in cost-sharing between industries and governments from the beginning, as this will likely impact long-term compliance.^56^ Indeed, ensuring buy-in from the sectors most exposed to ETs will be key to fostering quasi-voluntary compliance and avoiding political backlashes against enforcement, which might make reform attempts short-lived.^57^

Further complexity is introduced by the fact that specific measures, such as waste taxes or charges to avoid pollution from traffic congestions, will likely be better assessed and collected at the local rather than the national level.^58^ Indeed, local governments might be better placed to administer these taxes, as the most appropriate rate depends, for example, on the density of the urban population and their consumption behaviour (waste taxes), or the share of car ownership and peak driving hours (congestion charges). All of these characteristics are likely to vary across urban and rural settlements, making the introduction of a unified national rate less efficient. While being better placed to determine the appropriate set of these measures, local governments also have lower administrative capacity and different incentives to enforce taxes than central governments. In these cases, there could be scope for allowing revenue authorities to collect the revenue from these measures and then redistribute it back to local governments, but again, this gives rise to potential institutional conflict.^59^ Once more, this is without considering the case of federal states, in which much revenue-raising capacity lies with individual states, which could then end up having drastically different setups for the same tax.^60^

It must also be noted that there were situations in which the main obstacles identified were the lack of political will to implement ETs rather than of technical capacity,^61^ and situations in which the two were as important as each other,^62^ reflecting three broad dynamics.

First, ETs are seen as having the potential to depress industrial activities by adding to the cost of doing business, and increasing manufacturing capacity is seen as the main pathway out of poverty and to increase economic growth.^63^ Indeed, given that poverty reduction and employment generation remain the overarching priorities across SSA, there have been cases in which particular ministries have opposed the introduction or enforcement of strict environmental standards, which they saw as an unaffordable luxury.^64^

Second, ETs are widely perceived as regressive by the stakeholders interviewed,^65^ although there was a recognition that more information is required on who contributes most to different types of pollution before this could be determined.^66^ Some interviewees were especially worried about the impact of ETs on the cost of living in the short run, given the widespread lack of availability – or unaffordable price for the majority of the population – of green alternatives across domestic markets.^67^ Furthermore, this is compounded by the state actors’ somehow limited capacity to reach low-income households and less profitable firms, both of which will likely be affected by strict enforcement of ETs.^68^

Third, for both the above reasons, the introduction of ETs may potentially face sharp public resistance. This is mostly due to the capacity of elites to mobilise low-income households against measures that might be in the latter ultimate interests,^69^ and to the vocal opposition of industries accounting for significant shares of employment,^70^ making the argument for ensuring widespread buy-in before introduction even stronger.

## 5 Domestic appeal

Despite all of these obstacles, even in countries where there is currently little political support for a wider deployment of ETs, the situation is likely to change as the economy develops.^71^ Furthermore, there are countries where there is already widespread support for a more environmentally conscious development strategy, especially among the government and the elites.^72^ In others, pockets of support already exist, and opposition is not ubiquitous but rather concentrated in only a few sectors and ministries, so it should be reasonably straightforward to overcome it with backing from the top.^73^ In this context, it is then useful to understand how ETs could be made appealing to both political actors and the population at large.

Interestingly, there was some appreciation amongst the interviewees that ETs should be promoted as instruments whose main scope is addressing environmental issues, and not primarily generating more revenue.^74^ While the revenue contribution of particular measures might well be significant, stressing this aspect excessively might lead policymakers to deprioritise the environmental objective of ETs during policy formulation, as well as to decrease their appeal should revenue increases not materialise in the short term.^75^ Indeed, ETs were deemed more appealing to ministries of finance than to revenue authorities exactly for this reason – the latter are not likely to strongly push for the implementation of measures that will not help them meet revenue targets. On the other hand, ministries of finance have a wider mandate, which allows them to appreciate the contribution of ETs to national economic development.^76^ However, both institutions should be involved in their development; otherwise, there is a risk of developing measures that look great on paper but are impossible to administer.^77^

Furthermore, there was also widespread support for earmarking revenue from ETs to tackle environmental issues, as this will help to increase support from the general public and might contribute to fostering quasi-voluntary compliance.^78^ It must, however, also be noted that this was not a unitary position, as there are also countries in which ensuring budget fungibility is paramount so that any discussion about revenue earmarking might actually reduce ETs appeal amongst policymakers.^79^ On the other hand, the idea of using revenue from ETs to decrease taxation on capital or labour had virtually no support, as there is an overwhelming need to increase revenue across all SSA countries and taxes on capital and labour are not considered particularly high.^80^ In fact, this was seen as potentially counterproductive, as the contribution of ETs might well decrease over time if they have the intended behavioural effect, and were their revenue to be earmarked, it would lead to a decrease in the fungible budget for the government.^81^ However, in cases where some reforms of the income tax structure have already been considered by the government, it could be useful to introduce these in tandem with ETs, as this might help shore up support for the whole reform package.^82^

Some support for ETs might actually already be present amongst the wider population. This is both because there has been a visible increase in the incidence and impact of natural disasters in the last few years and because particular issues, such as pollution from traffic congestions and bad waste management in urban areas, are leading to increasing health costs.^83^ However, this support is likely to be quite differentiated around geographical lines, with ETs having much less traction in rural areas than they will have in urban ones. This is because demands for environmental action are likely to be strongly correlated with levels of education and because increasing the cost of exploitation of natural resources will be unpopular where they are an important source of livelihood.^84^ Hence, it will be relevant to understand where the government's political base is located to decide which measures to prioritise first, as creating momentum once ETs start to be introduced will be important for their successful and sustained implementation.

What must nonetheless be kept in mind is that, while environmental issues are becoming more salient for an increasing number of citizens, the demand for prompt action is still in its infancy. Hence, any positive environmental impact of ETs should be carefully weighed against their effect on employment creation and the cost of living, both of which remain a much higher priority for governments and citizens alike.^85^ Indeed, given the negative economic impact that the COVID-19 pandemic and the Russian invasion of Ukraine have had on SSA economies, it is unlikely that support will exist for the introduction of ETs with high associated tax rates in the short run.^86^ Hence, it will likely be better for ETs to first be introduced with low associated rates, which could then be increased in the future after widespread sensitisation campaigns coordinated with civil society organisations that could support these measures.^87^

## 6 Conclusions

The scope of this paper is to provide exploratory evidence on the type of institutional, administrative, and political obstacles to a wider implementation of ETs across SSA, as well as a few indications on how they might be made more appealing. Some of the study's results align with the limited existing literature, such as the lack of data on environmental issues and the technical capacity to analyse them, the limited impact and revenue contribution of existing measures or the necessity to consider the equity impact of ETs. On the other hand, the considerations elicited from our interviewees on fragmented policy processes, the dispersion of technical knowledge across MDAs and the lack of institutional coordination fora, as well as on the higher domestic appeal of discrete rather than overarching measures, have received very little attention in the literature up to this point. Given the general consensus among international financial institutions, multilateral development banks and the donor community that the deployment of ETs is a desirable goal, the hope is that this paper indicates areas where more research and engagement will be useful.

As it was shown, measures that could classify as ETs are present across all countries covered, ranging from fees on plastic products to levies on fossil fuels. However, most of these measures have been introduced to mobilise public funds rather than address environmental externalities. Despite this, quantifying their revenue contribution remains challenging, with only 17 countries across the continent reporting figures for their collection in 2021, which average 0.22 per cent of GDP (ATAF 2023). Understanding whether these measures are having any impact on the environment is equally hard, as they are seldom subjected to any rigorous evaluation, but the perception is that they are not particularly effective. Nonetheless, all stakeholders consider ETs as potentially useful instruments in tackling specific issues, such as pollution from solid waste and used cars, or excessive forest exploitation – when and if they are adequately devised.

However, much of the data necessary to develop effective ETs is currently lacking, from the amount and types of pollutants generated through industrial production to households’ willingness to pay for waste management. Given how costly reliable data is to collect, this is an area in which both donors and academia might consider providing support, the former by funding selected MDAs to improve data collection practices for ETs planning, and the latter by supporting the development of collection tools fit for LICs contexts. Attention would be best directed towards data that might facilitate the introduction of measures that are simple to administer, and both donors and research should focus on capacitating the relevant institutions to ensure that the data can be independently updated and analysed after initial collection. Similarly, investment is required to enable ministries of finance and revenue authorities to make use of this data, as the current analytical capacity in these institutions does not usually cover environmental issues. Some of this capacity might be present in EMAs, currently the most important player in the sector in all countries covered, so fostering cooperation between these MDAs might also be useful. Once this data is gathered, it should be used to assess whether the widespread concerns over the regressivity of different ETs are justified and, if so, devise administratively practical ways to offer support to low-income households and smaller firms that are disproportionally affected.

Promoting more effective coordination across MDAs will also be key to the success of ETs. This could start by carrying out an assessment of the existing mandates over natural resources and environmental assets of different government bodies, as well as whether their policy directives are synergistic or competitive. To be clear, this is far from a purely technical issue: deciding which resource use should be prioritised in cases of conflicting directives is an inherently political matter, and different MDAs should be expected to defend their current revenue-raising capacity. Hence, while international actors might be providing support on the initial stocktaking, having a clear direction from top political actors in the country will be required for any change to actually take place. The recent institution of ministries of the environment across various jurisdictions is a promising development in this regard, as it will contribute to bringing environmental issues into cabinet discussions, but it will be necessary to observe whether they can deliver on their mandate. The process of environmental policy coordination these ministries have been created for has already been identified as an area in need of further research (Bodin 2017), and while some evidence for advanced and transition economies is available (von Lüpke et al., 2023), these questions have not been explored in the context of LICs. This is a topic which should receive more scholarly attention, taking into the account both required mechanisms for coordination between central and local governments (Fuhr et al., 2018), as well as the conditions under which civil society organisations, both local and international, could support these processes (Bernauer et al., 2013).

Decisions will also be required on the use of the revenue from ETs. As discussed, there is a general lack of support for the idea of using this revenue to decrease taxes on capital and labour, so these arguments are better left alone. Conversely, earmarking this revenue to address environmental issues seems promising, although it would likely be more viable in contexts where governments are not under pressure to maintain the fungibility of the whole budget. Indeed, showing taxpayers that ET measures are directly contributing to addressing environmental issues will likely play an important role in fostering compliance, which should also be promoted through sensitisation and engagement with businesses whose reporting requirements will be affected. Civil society organisations could potentially play an important role in this regard and should be seen by governments as useful allies, but these organisations usually lack funding to carry out extended campaigns and engagement. This is an area where donors can provide some significant financial support, while academics can provide indications of what engagement strategies prove more impactful given a growing interest on civil society's role in fiscal matters.

Finally, future research should build on this exploratory study by addressing some of its current limitations. First, carbon taxes received little attention in this paper – this was not an explicit decision, but a consequence of the fact that they were barely mentioned by most interviewees. When they were, this was as a measure that had been given some consideration but not considered as a priority, given existing administrative capacity and data availability. While this situation might have changed since, given the passing of the EU carbon border adjustment mechanism, this shows that when considering the role of ETs in LICs, it is better to focus on issues perceived as priorities by local stakeholders than those preferred by the international community. Second, most countries covered were former British colonies in which common law represents the basis of the legal system. Hence, it will be of interest to assess whether both the obstacles to and the appeal of ETs are significantly different in Francophone and Lusophone African countries where civil law plays a much more significant role. As ETs deployment becomes more common, it will also be of interest to gather the perspective different from those of the purposedly selected actors of this study. Both civil society organisations and the private sector will play a role in hindering or promoting the success of environmental fiscal reforms in LICs, and their role warrants an explicit focus in future studies.

## CRediT authorship contribution statement

**Giovanni Occhiali:** Writing – original draft, Project administration, Methodology, Investigation, Formal analysis, Data curation, Conceptualization.

## Declaration of competing interest

No conflict of interest needs to be reported.

## Data Availability

No data was used for the research described in the article.

## Footnotes

1 After reviewing various definitions currently in use, Cottrell and Falcão (2018) proposes to define ETs as “*any compulsory, unrequited payment to general government imposed for an environmental reason and levied on a tax base that has a proven specific negative impact on the environment*”. The author finds this definition agreeable, as it allows to also cover payment which would normally qualify as “charges” or “fees”, but that are in essence governmental attempt to manipulate prices to influence behaviour with an environmental impact (Keen 2024).

2 Interview guidelines can be found in Appendix.1.

3 By the United Nations Development Program, see https://www.undp.org/africa.

4 By the World Bank, see https://datahelpdesk.worldbank.org/knowledgebase/articles/906519-world-bank-country-and-lending-groups.

5 While the exact features of carbon taxes varies across country, they can be generally defined as specific excise taxes usually levied on a ton – by weight or volume – of carbon dioxide equivalent (ATAF, 2022, which also discusses carbon taxation in Africa). It is also worth noting that what matters for policy purposes is the introduction of a price on carbon, which can also be achieved by setting up emission trading schemes – for a discussion on the differences behind the two strategies, see Parry et al. (2022).

6 For example, given current patterns of access to modern energy across SSA, carbon taxes will likely be progressive in most of their contexts, see Slunge and Sterner (2012) and Dorband et al. (2019).

7 See UNEP (2014a) for Ghana, UNEP (2014b) for Mauritius, UNEP (2015b) for Mozambique.

8 The analysis presented by OECD (2021) includes Cote d’Ivoire, Ghana, Kenya, Nigeria, and Uganda.

9 See Maino and Emrullahu (2022) for the impact of introducing carbon taxes in 20 fragile states in SSA.

10 See Advani et al. (2021).

11 See Holtz and Heitzig (2021) and Köhlin et al. (2021).

12 Interviews with the Rwanda Revenue Authority, the National Revenue Authority and Ministry of Finance of Sierra Leone, and the Uganda Revenue Authority.

13 Interviews with the Rwanda Revenue Authority and the National Revenue Authority of Sierra Leone.

14 Interviews with the Rwanda Revenue Authority and the National Revenue Authority and Ministry of Finance of Sierra Leone.

15 Interviews with the Ministry of Finance of Ethiopia, the Ministry of Finance of Sierra Leone, and an independent PFM consultant in Uganda.

16 Interviews with the eSwatini Revenue Authority, the Ethiopian Ministry of Finance, the Ministry of Finance and the National Revenue Authority of Sierra Leone and the Uganda Revenue Authority.

17 p.m.2.5 stands for “particulate matter of a diameter equal or smaller than 2.5 μm”, which mostly originates from human activities related to fossil fuel combustion, see WHO 2023.

18 Interviews with the eSwatini Revenue Authority, the Ethiopian Ministry of Finance and the Nigerian Governors Forum.

19 Interviews with the eSwatini Revenue Authority, the Ethiopian Ministry of Finance, and the National Revenue Authority and the Ministry of Finance of Sierra Leone.

20 Interviews with the eSwatini Revenue Authority, the Ethiopian Ministry of Finance, the Uganda Revenue Authority and the Ministry of Finance of Uganda.

21 Interviews with the Ethiopian Ministry of Finance, the Rwanda Revenue Authority, and the Uganda Revenue Authority.

22 Interviews with the Rwanda Revenue Authority, and the National Revenue Authority and the Ministry of Finance of Sierra Leone.

23 Interviews with the Ethiopian Ministry of Finance, the Nigerian Governors Forum, and the Uganda Revenue Authority.

24 Interviews with the eSwatini Revenue Authority, the Ethiopian Ministry of Finance, and the National Revenue Authority of Sierra Leone.

25 Interviews with the Ethiopian Ministry of Finance and the National Revenue Authority of Sierra Leone.

26 Interviews with the Ethiopian Ministry of Finance, the Rwanda Revenue Authority, the National Revenue Authority of Sierra Leone, and the Ugandan Revenue Authority.

27 Interview with the National Revenue Authority of Sierra Leone.

28 Interviews with the Nigerian Governors Forum, the Rwanda Revenue Authority, the National Revenue Authority and the Ministry of Finance of Sierra Leone, and the Uganda Revenue Authority.

29 Interviews with the eSwatini Revenue Authority and the Ethiopian Ministry of Finance. It is also worth noting that the lack of cross-ministries engagement on fiscal matters across SSA is a wider issue not limited to environmental taxation – see, for example, Elliott et al. (2022) for health and Hilson (2020) for mining.

30 Interviews with the Ethiopian Ministry of Finance and the National Revenue Authority of Sierra Leone.

31 Interviews with the eSwatini Revenue Authority, the Rwanda Revenue Authority, and the Uganda Revenue Authority.

32 Interviews with the National Revenue Authority of Sierra Leone and the Uganda Revenue Authority.

33 Interviews with the Ethiopian Ministry of Finance and the National Revenue Authority of Sierra Leone.

34 Interviews with the eSwatini Revenue Authority, the Rwanda Revenue Authority, and the National Revenue Authority of Sierra Leone.

35 Interviews with the National Revenue Authority and the Ministry of Finance of Sierra Leone.

36 Interview with the Rwanda Revenue Authority.

37 Interviews with the Nigeria Governors Forum, the Rwanda Revenue Authority, the National Revenue Authority and the Ministry of Finance of Sierra Leone, and the Uganda Revenue Authority.

38 Interviews with the eSwatini Revenue Authority, the National Revenue Authority and the Ministry of Finance of Sierra Leone, the Uganda Revenue Authority, the Ministry of Finance of Uganda, and an independent PFM consultant from Uganda.

39 Interviews with the National Revenue Authority of Sierra Leone and the Ministry of Finance of Uganda.

40 Interview with the Ethiopian Ministry of Finance.

41 Interviews with the eSwatini Revenue Authority, the Ministry of Finance of eSwatini, the Ministry of Finance of Ethiopia, the Rwanda Revenue Authority, the National Revenue Authority, the Ministry of Finance of Sierra Leone, the Uganda Revenue Authority, and the Ministry of Finance of Uganda.

42 Interviews with the Rwanda Revenue Authority, the Uganda Revenue Authority, and the Ministry of Finance of Uganda.

43 Interviews with the eSwatini Revenue Authority and the Ministry of Finance of Ethiopia.

44 Interview with the Uganda Revenue Authority.

45 Interviews with the eSwatini Revenue Authority and the Uganda Revenue Authority.

46 Interviews with the Rwanda Revenue Authority, the Ministry of Finance of Sierra Leone and the Uganda Revenue Authority.

47 Interviews with the eSwatini Revenue Authority, the Ministry of Finance of Ethiopia and the National Revenue Authority of Sierra Leone.

48 Interview with the Uganda Revenue Authority.

49 Interview with the National Revenue Authority of Sierra Leone.

50 Interview with the National Revenue Authority of Sierra Leone.

51 Interview with the Rwanda Revenue Authority and Uganda Revenue Authority.

52 It is worth noting here that some of the considerations made by the interviewees mirror those expressed with regard to VAT introduction in LICs in the 1990s and 2000s. While VAT ended up being adopted in over 160 countries, contributing a significant share of revenue in most of them, the complexity of its administration indeed remains one of the main obstacles preventing the delivery of all its theoretical benefits (Mascagni et al., 2023).

53 Interviews with the Ministry of Finance of Ethiopia, the Ministry of Finance of Sierra Leone, and the Uganda Revenue Authority.

54 Interviews with the eSwatini Revenue Authority, the Ministry of Finance of eSwatini, and the National Revenue Authority of Sierra Leone.

55 Interviews with the National Revenue Authority of Sierra Leone and the Uganda Revenue Authority.

56 Interviews with the eSwatini Revenue Authority and the Ministry of Finance of eSwatini.

57 Interviews with the Ministry of Finance of Ethiopia and the National Revenue Authority of Sierra Leone.

58 Interviews with the Nigerian Governors Forum, the National Revenue Authority of Sierra Leone, and the Uganda Revenue Authority.

59 Interviews with the eSwatini Revenue Authority, the National Revenue Authority of Sierra Leone, and the Uganda Revenue Authority.

60 Interviews with the Ministry of Finance of Ethiopia and the Nigerian Governors Forum.

61 Interview with the Nigerian Governors Forum.

62 Interview with the Ministry of Finance of Ethiopia.

63 Interview with the Nigerian Governors Forum.

64 Interviews with the Ministry of Finance of Ethiopia, the Nigerian Governors Forum, and the National Revenue Authority of Sierra Leone.

65 Interviews with the eSwatini Revenue Authority, the Ministry of Finance of eSwatini, the Rwanda Revenue Authority, and the Uganda Revenue Authority.

66 Interviews with the National Revenue Authority and the Ministry of Finance of Sierra Leone.

67 Interviews with the Rwanda Revenue Authority, and the National Revenue Authority and the Ministry of Finance of Sierra Leone.

68 Interviews with the Revenue Authority and the Ministry of Finance of Sierra Leone, and the Uganda Revenue Authority.

69 Interview with the Ministry of Finance of Sierra Leone.

70 Interview with the Ministry of Finance of Ethiopia.

71 Interview with the Ministry of Finance of Ethiopia.

72 Interviews with the eSwatini Revenue Authority, the Ministry of Finance of eSwatini, the Rwanda Revenue Authority, the Uganda Revenue Authority and the Ministry of Finance of Uganda.

73 Interviews with the Ministry of Finance of Sierra Leone and with an independent PFM consultant in Uganda.

74 Interviews with the eSwatini Revenue Authority, the National Revenue Authority and the Ministry of Finance of Sierra Leone, and the Uganda Revenue Authority.

75 Interviews with the eSwatini Revenue Authority, the National Revenue Authority and the Ministry of Finance of Sierra Leone.

76 Interviews with the Ministry of Finance of Sierra Leone and the Uganda Revenue Authority.

77 Interview with the Uganda Revenue Authority.

78 Interviews with the Ministry of Finance of eSwatini, the Ministry of Finance of Ethiopia, and the National Revenue Authority and the Ministry of Finance of Sierra Leone. It must also be noted that this consideration widely resonates with research carried out in HICs, see Pirttilä (1999), Brett and Keen (2000) and Kallbekken and Aasen (2010).

79 Interview with the Ministry of Finance of Uganda.

80 Interviews with the eSwatini Revenue Authority, the Ministry of Finance of eSwatini, the Ministry of Finance of Ethiopia, the National Revenue Authority and the Ministry of Finance of Sierra Leone, the Uganda Revenue Authority, and an independent PFM consultant in Uganda.

81 Interviews with the eSwatini Revenue Authority, the Ministry of Finance of eSwatini, the Ministry of Finance of Ethiopia, the National Revenue Authority and the Ministry of Finance of Sierra Leone.

82 Interviews with the Ministry of Finance of Ethiopia, the Rwanda Revenue Authority, the Ministry of Finance of Sierra Leone and the Uganda Revenue Authority.

83 Interviews with the Nigerian Governors Forum, the Rwanda Revenue Authority, the National Revenue Authority and the Ministry of Finance of Sierra Leone, and the Uganda Revenue Authority.

84 Interviews with the Nigerian Governors Forum and with the Uganda Revenue Authority.

85 Interviews with the eSwatini Ministry of Finance, the Nigerian Governors Forum, and the Uganda Revenue Authority.

86 Interviews with the eSwatini Revenue Authority, the National Revenue Authority and the Ministry of Finance of Sierra Leone, and the Uganda Revenue Authority.

87 Interviews with the Nigerian Governors Forum, the Ministry of Finance of Sierra Leone and the Uganda Revenue Authority.
