# Supplementary material for: Obstacles and appeal of environmental taxation: Insights from sub-Saharan Africa
Source: Environ Dev. 2024 Sep;51:101037. doi: 10.1016/j.envdev.2024.101037 (PMC11385670; doi:10.1016/j.envdev.2024.101037)
Supplement: Multimedia component 1 [file mmc1.docx]

**Appendix.1**

**Interview Guidelines**

**I. Assessing environmental issues and solutions**

1. Is there any environmental problem affecting the country whose solution is considered a top priority by the government?
   1. [IF YES] What policies are currently in place to tackle this, and do they involve a market-based instrument, including – but not exclusively – tax measures?
      1. [IF YES] Can you please give us some details of these measures?
      2. [IF YES] What MDAs were involved in its formulation?
   2. [IF YES] How long will it take for these policies to deliver the expected results, and have they been costed over this period?
   3. [IF NO] Is there any environmental issue which you think could become a problem if left unchecked?
      1. [IF YES] Do you think its solution should be prioritised, and if yes, why do you think this has not happened?

**II. Assessing current fiscal measure which might have an environmental impact.**

1. What national or local taxes currently in place target a tax base with an environmental connection? *(These could be anything connected with pollution, waste and/or extraction of resources, especially renewables)*
   1. [IF THERE ARE SOME] Were some of these introduced with an explicit environmental goal, or mostly as revenue measures?
      1. [IF NOT ENVIRONMENTAL] Do you think that these taxes could be remodulated to increase their environmental effectiveness?
   2. [IF THER ARE SOME] When were these measures introduced, and how often have they been revised since? Are there clauses for pegging of rates to inflation or for regular revisions?

**III. Assessing the connection between environmental protection framework and fiscal policy.**

1. What existing government ministries, department and agencies are in charge of setting environmental standards and regulations?
2. Are there institutional fora for these MDAs to interact amongst each other and with the revenue authority should they wish to coordinate the role of tax policies in environmental protection?
   1. [IF THERE ARE SOME] What is the explicit goal of these meetings? How often are they convened? Who are the participants? Are there some players who hold more power than other?
   2. [IF THERE ARE SOME] Do you think that the current institutional structure of these meetings is fit-for-purpose?
      1. [IF NO] What would you change?
   3. [IF THERE ARE NONE] Do you think that it would be useful to have such fora in place?
      1. [IF YES] What obstacles do you think exist for their establishment? Have they ever been proposed?
      2. [IF NO] Why not?
3. In your opinion, how effective are current environmental regulations and standard? Please, explain your answer.

**IV. Environmental fiscal reform – domestic appeal.**

1. In your opinion and from your knowledge of other jurisdictions, are there environmental issues in your country which could be reduced by the introduction of a specific fiscal instruments?
   1. [IF YES] Which one, and what type of tax measure do you think would help? *(I.e. excises, royalties, fees, etc.)*
   2. [IF YES] Do you think that the devising such fiscal measures has different modelling and data requirements than those currently under administration?
      1. [IF YES] Do you think that the technical capacity and data to devise such policies exists across different government MDAs?
         1. [IF NOT] What would be the best strategy to acquire such capacity?
   3. [IF YES] Do you think that they would be progressive or regressive, and why?
      1. [IF REGRESSIVE] Do you think that there could be way to balance the regressivity? Please, explain your answer. (*the “how” if yes especially)*
   4. [IF YES] Do you think that they would have a relevant revenue contribution?
      1. [IF A FIGURE IS GIVEN] Could you tell us the reasoning behind that figure?
      2. [IF NO] Do you think that the lack of revenue aspect makes it less appealing to discuss or administer?
   5. [IF YES] At which level of government (central/local) and by which MDAs do you think that they should be administered, and why?
   6. [IF YES] Do you think that they would require different administrative arrangements from others currently under administration, and if so, why?
      1. [IF YES] Do you think that they would be more costly to comply with than other existing measures?
      2. [IF YES] Do you think that enforcement capacity is present to ensure compliance?
2. How politically attractive are fiscal measures as a potential solution to environmental issues amongst government stakeholders? Please explain your answer.
   1. Do you think that earmarking revenue from the tax for a specific use might make it more attractive? If so, which use, and would someone resist this?
   2. Do you think that reducing other taxes – PAYE and CIT particularly – could contribute to make these taxes more attractive?
3. Do you think that there is a political demand across some sector of the population for stronger government action on environmental issues?
   1. [IF YES] Do you think that this demand is strong enough to be leveraged for increased taxation?

**VI. Carbon taxes [ONLY IF THEY HAVE NOT BEEN PART OF THE CONVERSATION UP TO THIS POINT.]**

1. If your opinion, would carbon taxes be a useful policy tool in your country context? Explain your answer.
2. Has there been any discussion about carbon taxes in the government?
   1. [IF YES] Can you give us an idea about what are the government plans in this regard?
   2. [IF YES] How likely do you think it is that the measure will be pushed ahead? Please explain your answer.
      1. [IF LIKELY] How long do you think it will take to implement it?
